# Neutrophil FcγRIIA availability is associated with disease activity in systemic lupus erythematosus

**DOI:** 10.1186/s13075-020-02221-z

**Published:** 2020-05-29

**Authors:** Anders A. Bengtsson, Helena Tyden, Christian Lood

**Affiliations:** 1 Division of Rheumatology, Department of Clinical Sciences Lund, Lund University, Lund, Sweden; 2 Division of Rheumatology, Department of Medicine, University of Washington, 750 Republican Street, Room E-545, Seattle, WA 98109 USA

**Keywords:** Systemic lupus erythematosus, Immune complex, Nephritis, Interferon, Biomarker

## Abstract

**Background:**

Immune complexes (ICs) are detectable in a variety of inflammatory diseases, including systemic lupus erythematosus (SLE), reflecting autoantibody binding to antigens. Though ICs are the main contributors to disease pathogenesis through FcγR-mediated inflammation and organ damage, IC levels are not part of the clinical assessment of SLE. The aim of this study was to explore the clinical utility of analyzing levels of ICs in SLE patients using a novel technology, IC-FLOW.

**Methods:**

Paired serum samples, at the time point of high and low disease activity (*n* = 92), were analyzed using two assays: an IC ELISA from a commercial company and a novel in-house flow cytometry-based method, IC-FLOW. IC-FLOW measures FcγRIIA availability on the neutrophil cell surface by flow cytometry, whereas the commercial ELISA measures IC binding to C1q.

**Results:**

Using IC-FLOW, 90% of SLE patients with active disease had elevated levels of circulating ICs (*p* < 0.0001). Using the commercial assay, only 17% of SLE patients had elevated levels of circulating ICs. For both assays, levels of ICs reflected active disease as determined by SLEDAI (*r* = 0.45, *p* < 0.0001) and were associated with type I IFN activity (*r* = 0.37, *p* = 0.001), and complement consumption (*p* = 0.0002). Levels of ICs measured with IC-FLOW, but not with the commercial ELISA, were associated with active lupus nephritis (*p* = 0.004).

**Conclusions:**

This novel FcγRIIA-IC assay can detect levels of circulating ICs in patients with SLE. Analyzing IC levels may facilitate monitoring of disease activity, as well as identify patients at risk of lupus nephritis, allowing for early preventive interventions.

## Introduction

Circulating immune complexes (ICs), formed upon recognition of antigens by antibodies, are detectable in a variety of systemic diseases, including autoimmune and infectious diseases. In systemic lupus erythematosus (SLE), an autoimmune rheumatic disease, ICs deposit in tissue, including skin and kidney, to induce complement activation and organ damage. Further, ICs are engulfed by immune cells causing inflammation including induction of type I interferons (IFNs) [1, 2], as well as the formation of neutrophil extracellular traps (NETs) [3, 4]. Although central to the disease pathogenesis, levels of circulating ICs are rarely measured in reference laboratories due to the lack of specificity in current assays with a broad array of assay measuring different features of ICs [5]. Early studies detected circulating ICs by their capacity to bind to neutrophils as determined by immunofluorescence techniques [6–8]. Current technologies, however, primarily rely on the capacity of circulating ICs to either fix complement C1q or bind complement C3 antibodies and/or precipitate using polyethylene glycol (PEG) [5]. Comparing C1q-based ELISAs, the overall agreement was about 50% between four commercially available assays [9]. The main concerns relate primarily to the ability of anti-C1q antibodies and rheumatoid factor (e.g., anti-IgG antibodies) to interfere with the ELISAs. Finally, though complement opsonization is an important event in the clearance of ICs, complement opsonization, as shown by us and others, neutralizes the ICs, reducing their inflammatory capacity [2, 10]. Thus, assessing complement-bearing ICs will primarily target non-inflammatory ICs and not the harmful ICs. The inflammatory trigger relies on ICs engaging FcγRs on immune cells through the Fc portion of the IgG molecule.

We recently found that FcγRIIA is the main FcγR responsible for the uptake of ICs by neutrophils [3]. In the current study, we investigated whether our novel technology, IC-FLOW, assessing the availability of FcγRIIA on a neutrophil cell surface, could provide a novel, clinically meaningful method of assessing levels of circulating ICs in SLE. Several methods have been suggested for the analysis of circulating ICs, including PEG-dependent precipitation, binding of ICs to C1q-coated plates, or binding of ICs to anti-C3 antibodies [5]. We here demonstrate that the analysis of neutrophil FcγRIIA cell surface expression, and not IgG levels, by flow cytometry accurately captures levels of circulating ICs in patient samples. The advantages of our novel technology are the lack of influence from rheumatoid factor and anti-C1q antibodies and the assessment of “pathogenic” ICs, e.g., ICs signaling through inflammatory FcγR-mediated pathways rather than through complement receptors.

## Materials and methods

### Patients

SLE patients (*n* = 92), recruited at the Skåne University Hospital, Lund, Sweden, were retrospectively selected from our biobank with stored samples to include patients with matched samples at time points of both high and low disease activity. Healthy controls (*n* = 100) were recruited at the Skåne University Hospital, Lund, Sweden. The study was approved by the regional ethics boards (LU06014520 and LU 378-02). Informed written consent was obtained from all participants according to the Declaration of Helsinki. The patient cohort is presented in Table 1 and has been described in great detail previously [12–18]. Nephritis was defined as the new onset of urinary casts, hematuria, pyuria, and/or proteinuria according to SLEDAI-2 K [11].

**Table 1:** Patient characteristics

Cohort	SLE high	SLE low	HC
Individuals, no.	92	92	100
Age (median, range)	42 (14–73)	44 (19–81)	49 (16–81)
Disease duration (median, range)	4 (0–40)	8 (0–43)	N/A
Gender (% female)	89	89	85
Ethnicity (% white)	90	90	90
SLEDAI (median, range)	8 (2–28)	2 (0–12)	N/A
Active nephritis^1^ (%)	31	1	N/A
Nephritis ever (%)	43	43	N/A
class I (%)	0	0	N/A
class II (%)	0	0	N/A
class III (%)	6	6	N/A
class IV (%)	76	76	N/A
class V (%)	18	18	N/A
Anti-C1q antibodies current (%)	27	24	N/A
Immunosuppressive treatment (%)	40	50	N/A
Azathioprine (%)	25	25	N/A
CellCept (%)	18	17	N/A
Methotrexate (%)	2	0	N/A
Rituximab (%)	0	0	N/A
Cyclosporine (%)	9	9	N/A
Prednisone treatment (%)	68	68	N/A
Prednisone dose (median, range)	10 (0–40)	10 (0–40)	N/A
Hydroxychloroquine (%)	30	37	N/A

^1^Defined as new onset of urinary casts, hematuria, pyuria and/or proteinuria according to SLEDAI-2 K [11]

### Type I interferon assay

Type I IFN activity was measured as previously described assessing the capacity of circulating type I IFNs to signal through IFNAR [19–21]. Briefly, WISH cells were cultured with patient serum (50%) and analyzed for the induction of six IFN-regulated genes (*LY6E*, *MX1*, *OAS1*, *ISG15*, *IFIT1*, *EIF2AK2*) and three housekeeping genes (*GAPDH*, *PPIB*, *B2M*) using the Quantigene Plex 2.0 assay as described by the manufacturer (Panomics, Inc.). Increased type I IFN activity was defined as a 2-fold increase in type I IFN-regulated genes as compared to healthy controls.

### Autoantibodies and complement components

Autoantibodies, including dsDNA (*Crithidia luciliae* immunofluorescence test) and complement levels in the serum, were measured according to routine analyses at the Department of Clinical Immunology, Skåne University Hospital, Lund, Sweden, performed at the Department of Laboratory Medicine, Lund, Sweden.

### Immune complex assays

Levels of immune complexes were analyzed using an in-house method, IC-FLOW. Briefly, neutrophils were isolated through density gradient (Polymorphprep, Axis-Shield) and incubated with sera (10%) for 90 min in RPMI-1640 medium. After serum incubation, and potential IC-binding and internalization of FcγRIIA, neutrophils were analyzed for cell surface expression of FcγRIIA by flow cytometry using FITC-conjugated IV.3 (STEMCELL Technologies) and PE-conjugated FUN-2 (BioLegend) antibody clones. The results are presented as micrograms per milliliter using heat-aggregated IgG of known concentration as a standard curve. For the commercial ELISA, levels of ICs were analyzed as per the company’s instructions (Quidel).

### Statistics

For non-paired sample sets with non-Gaussian distribution, the Mann-Whitney *U* test and Spearman’s correlation test were used, as applicable. In some analyses, logistic regression analysis was used for dichotomized variables. As a cutoff for positivity, the 95th percentile of the healthy controls was used. GraphPad Prism and IBM SPSS were used for the analyses. All analyses were considered statistically significant at *p* < 0.05.

## Results

### FcγRIIA internalization is a marker of IC binding

**Fig. 1:**
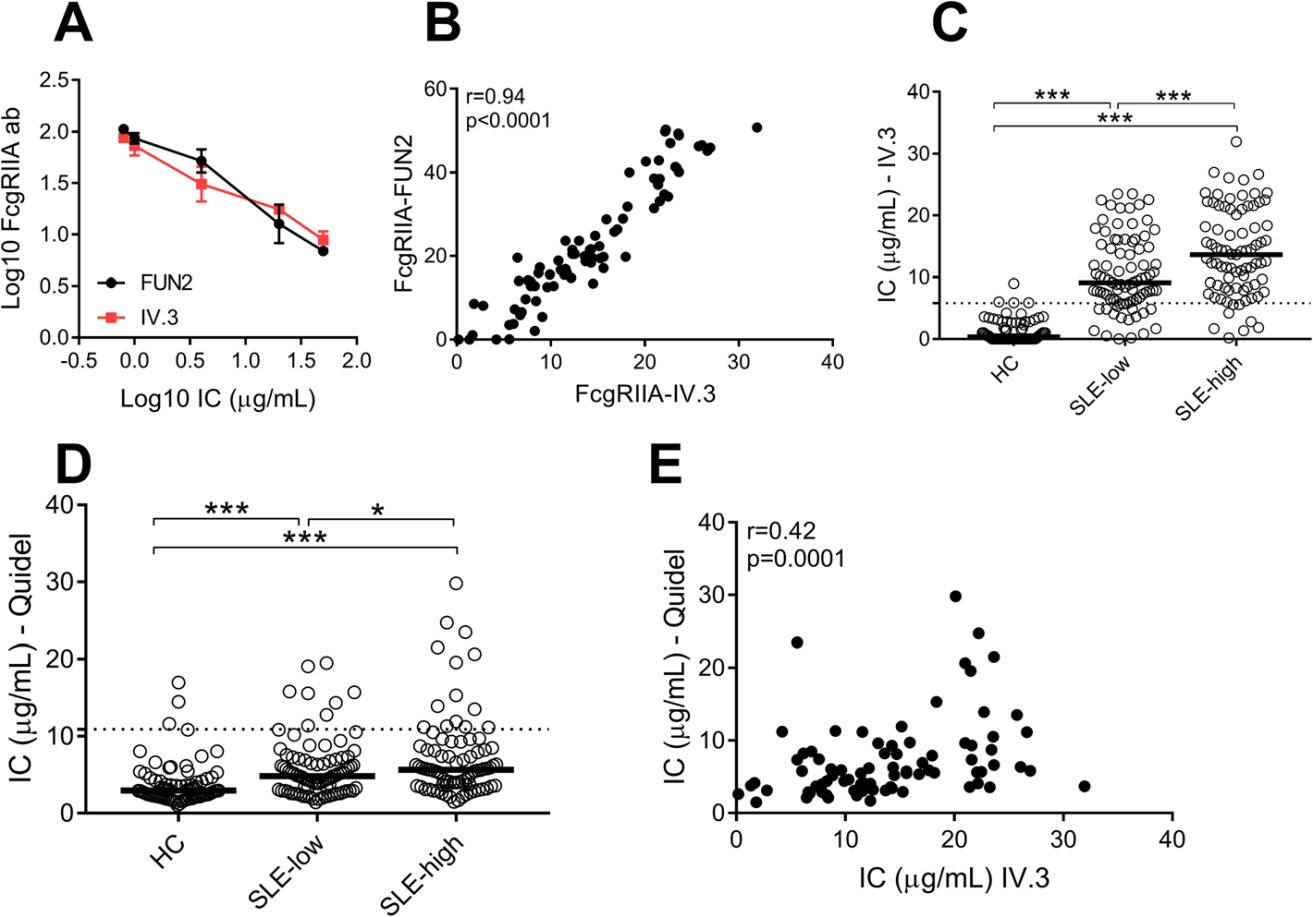
Development of a novel assay for the detection of circulating immune complexes. **a** Levels of FcγRIIA on neutrophils upon addition of heat-aggregated IgG (IC) as determined by binding of FcγRIIA antibody clones IV.3 (red) and FUN-2 (black). **b** Correlation plot for IV.3 and FUN-2. **c**, **d** Levels of ICs in healthy individuals (HC) and patients with SLE at the time point of low and high disease activity as measured by **c** IV.3 and **d** commercial assay. **e** Correlation plot for the two IC assays. Statistical analyses were performed using the Mann-Whitney *U* test and Spearman’s correlation with *p < 0.05,**p < 0.01, and ***p < 0.001

We recently found that FcγRIIA was the main FcγR involved in the uptake of circulating ICs by neutrophils [3]. Upon binding of ICs, the IgG-binding domain of FcγRIIA is occupied, and the receptor is subsequently internalized. Consistently, upon addition of heat-aggregated IgG (HAGG) to neutrophils, we found a dose-dependent reduction in the levels of cell surface FcγRIIA as determined by flow cytometry using two distinct FcγRII antibody clones: IV.3 and FUN-2 (Fig. 1a). Of note, the two FcγRII antibodies strongly correlated (*r* = 0.94, *p* < 0.0001, Fig. 1b), suggesting that they detect the same process, i.e., binding of ICs to FcγRIIA.

### SLE patients have elevated levels of circulating ICs

After establishing that the assay, IC-FLOW, was able to quantify levels of circulating ICs in an FcγRIIA-dependent manner, we next asked whether patients with SLE had elevated levels of ICs. As determined by IV.3 staining, levels of circulating ICs were highly elevated in SLE patients as compared to healthy individuals (*p* < 0.0001, Fig. 1c). Similar to our in-house assay, elevated levels of ICs were found in SLE patients also with the commercial assay (*p* < 0.0001, Fig. 1d), consistent with what has been described for C1q- and C3d-based ELISAs [22, 23]. The two assays only partially correlated (*r* = 0.42, *p* < 0.0001, Fig. 1e). Using the 95th percentile of healthy controls as a cutoff, 90% of SLE patients had elevated levels of circulating IC at the time point of active disease as determined by the IC-FLOW technology. In contrast, only 17% of the SLE patients were positive using the commercial ELISA. Similar results were seen also for patients with low disease activity (78% vs 9%, respectively, for the different assays).

### Levels of circulating immune complexes are associated with disease activity

**Fig. 2:**
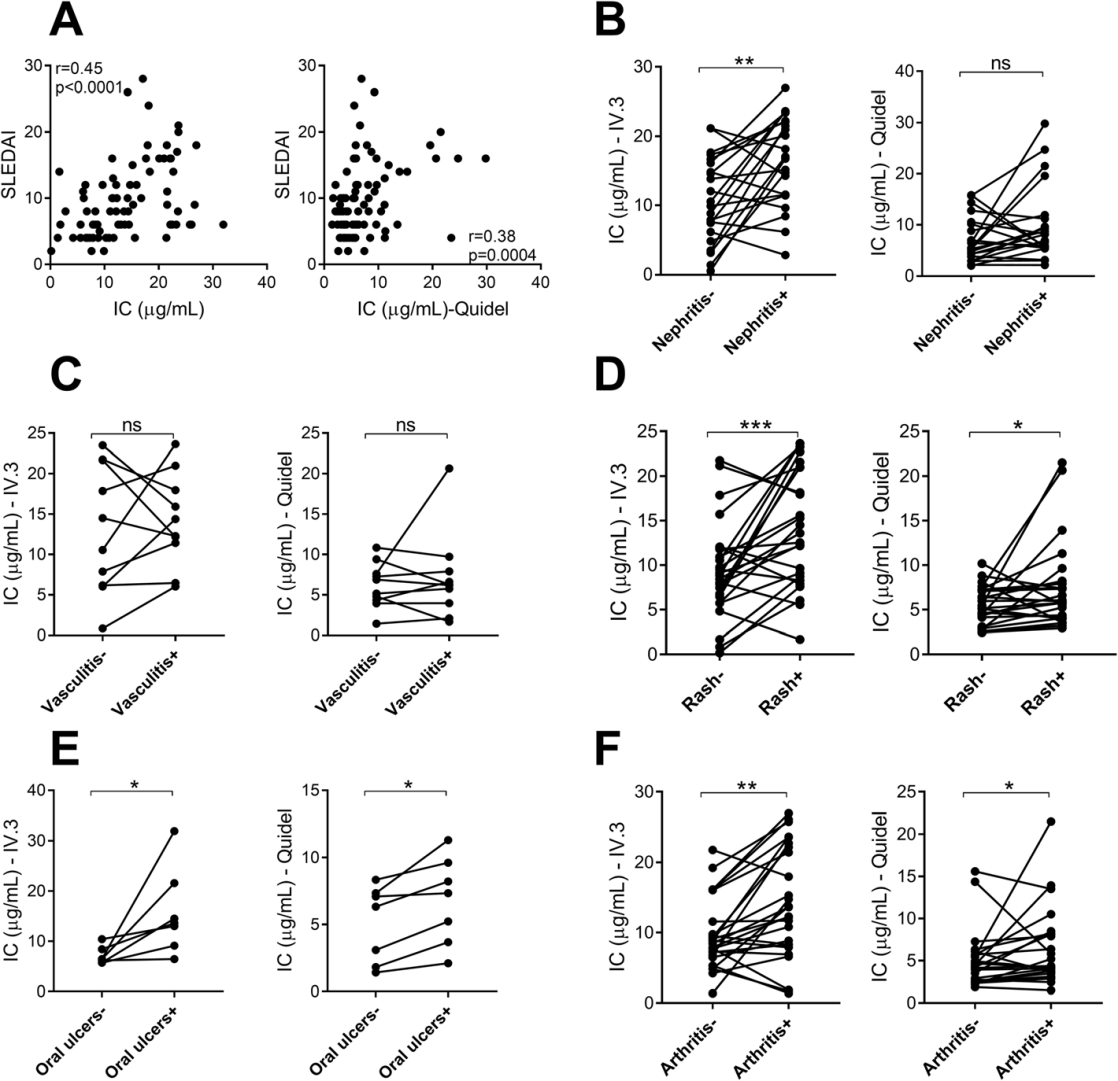
Association between levels of circulating immune complexes and disease activity. **a** Levels of circulating immune complexes were correlated with disease activity, SLEDAI. **b**–**f** Levels of ICs were analyzed in patients with (+) or without (−) active **b** nephritis, **c** vasculitis, **d** rash, **e** oral ulcers, and **f** arthritis. Statistical analyses were performed using the Mann-Whitney *U* test and Spearman’s correlation with *p < 0.05,**p < 0.01, and ***p < 0.001

**Fig. 3:**
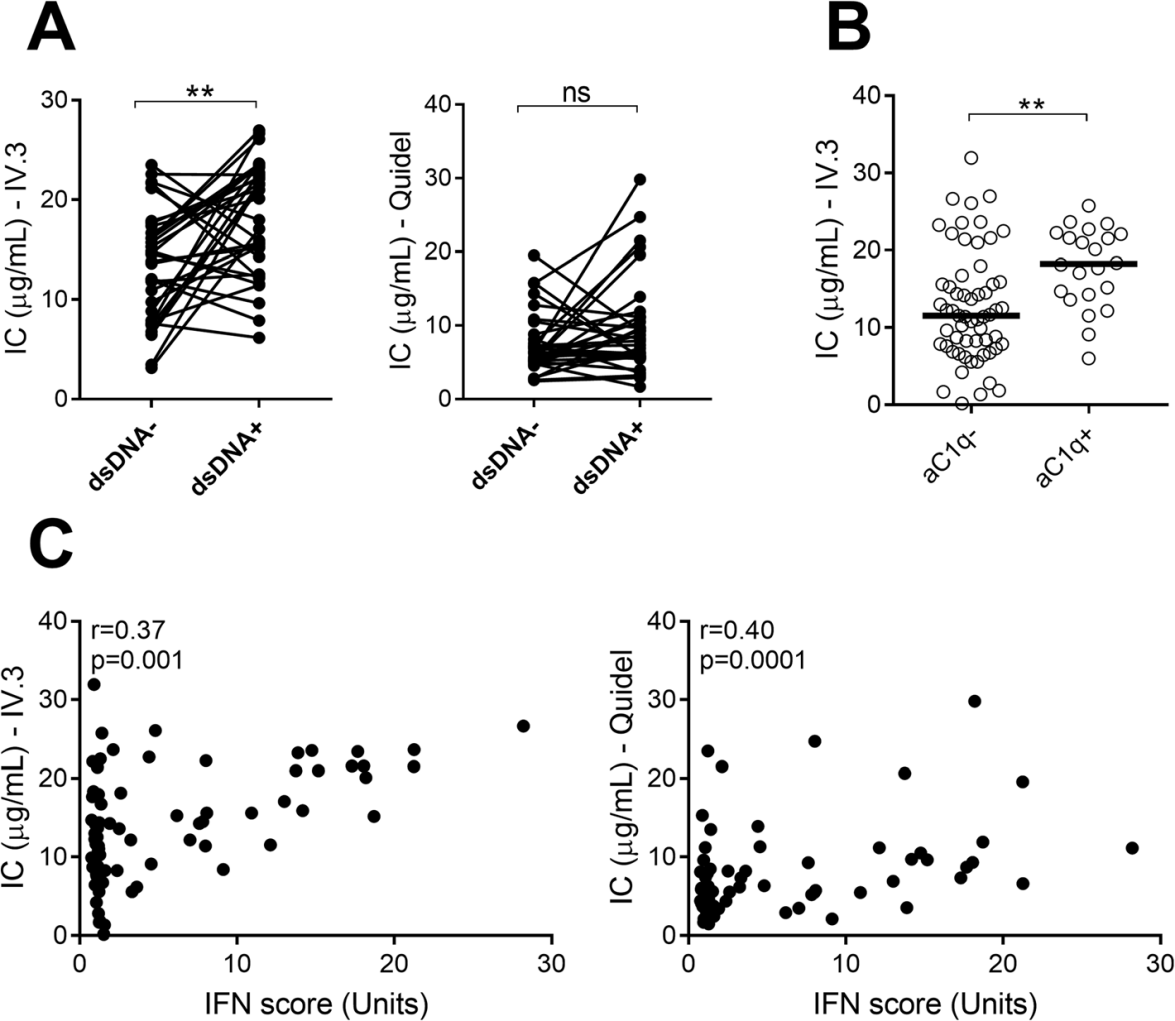
Levels of circulating immune complexes are associated with autoantibodies and type I IFNs. **a** Levels of circulating ICs, analyzed by both IC-FLOW and commercial ELISA, were compared between time points of the absence or presence of anti-dsDNA antibodies. **b** Levels of circulating ICs were analyzed in patients with high disease activity and stratified based on the presence of anti-C1q antibodies (aC1q). **c** Levels of ICs were correlated with type I IFN activity score. Statistical analyses were performed using the Mann-Whitney *U* test and Spearman’s correlation with *p < 0.05,**p < 0.01, and ***p < 0.001

As demonstrated in Fig. 1c, d, levels of circulating ICs were elevated in patients with active disease as compared to patients with low disease activity. Further, levels of circulating ICs correlated with SLEDAI at the time point of active disease as measured by IC-FLOW as well as the commercial assay (*r* = 0.45, *p* < 0.0001; *r* = 0.38, *p* = 0.0004, respectively, Fig. 2a). ICs are commonly found deposited in a tissue, including joints, skin, and kidney, promoting local inflammation and organ damage in SLE patients. Given the association between levels of circulating ICs and disease activity, we next asked whether levels of circulating ICs would reflect disease activity in specific organ systems, including the skin, joints, and kidneys. In contrast to early studies [24, 25], comparing paired patient samples at the time point of high disease activity with corresponding patient samples at the time point of low disease activity, levels of circulating ICs were found to be elevated in patients with the following organ manifestations: nephritis, rash, oral ulcers, and arthritis (*p* = 0.004, *p* = 0.0007, *p* = 0.02, and *p* = 0.002, respectively, Fig. 2) as compared to patients without those disease manifestations. Patients with vasculitis did not have statistically significant elevated levels of IC (Fig. 2c). In contrast to IC-FLOW, the commercial ELISA could not distinguish between active and inactive nephritis (Fig. 2b). Further, levels of ICs were associated with the presence of anti-C1q antibodies, known to be associated with renal disease (Fig. 3b).

### Levels of ICs are associated with immunopathological markers of disease

**Fig. 4:**
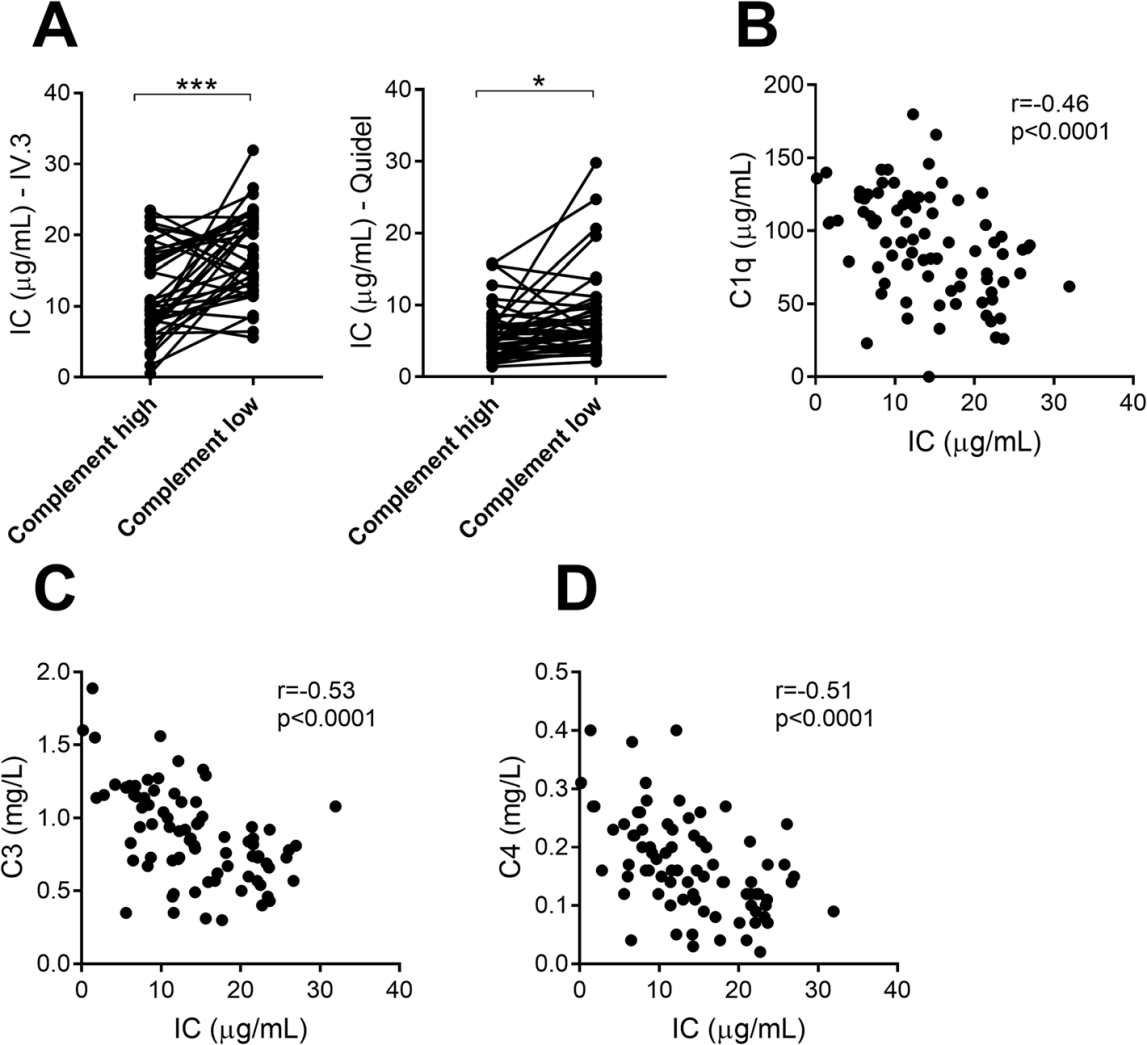
Levels of immune complexes are associated with complement consumption. **a** Levels of circulating ICs, analyzed by both IC-FLOW and commercial ELISA, were compared between time points of low or high complement C3/C4 levels. **b**–**d** Levels of circulating ICs were correlated with levels of **b** complement C1q, **c** complement C3, and **d** complement C4 in patients with high disease activity. Statistical analyses were performed using the Mann-Whitney *U* test and Spearman’s correlation with *p < 0.05 and ***p < 0.001

Asking whether levels of ICs were associated with serological markers of disease activity, we found that IC-FLOW, but not the commercial ELISA, was associated with the presence of anti-dsDNA antibodies (Fig. 3a). We have shown that nucleic acid-containing ICs are potent inducers of type I IFN production by plasmacytoid dendritic cells in vitro [2]. However, whether levels of ICs in vivo would relate to ongoing type I IFN activity in SLE patients is not known. As shown in Fig. 3c, type I IFN activity correlated with levels of circulating ICs as measured by both assays (Fig. 3c). ICs, both circulating and tissue-deposited, are potent activators of the classical pathway of the complement system. Consistently, levels of ICs were associated with complement consumption for both assays (*p* = 0.0002 and *p* = 0.01, respectively, Fig. 4a). Further, levels of ICs were inversely correlated with complement C1q, C3, and C4 (*r* = − 0.48, *p* < 0.0001; *r* = − 0.53, *p* < 0.0001; and *r* = − 0.51, *p* < 0.0001, respectively, Fig. 4b–d).

## Discussion

Immune complexes play a central role in many autoimmune diseases, contributing to inflammation and organ damage often through FcγR-mediated mechanisms. Though an abundance of assays has been developed to quantify levels of circulating ICs, they have failed in their specificity as well as due to technical properties, including interactions with autoantibodies such as rheumatoid factor and anti-C1q antibodies. In the current study, we propose that analyzing IC binding to FcγRIIA using flow cytometry may be a novel and superior approach to assess levels of inflammatory and pathogenic IC relevant to disease progression. Several methods have been suggested for the analysis of circulating ICs, including PEG-dependent precipitation, binding of ICs to C1q-coated plates, or binding of ICs to anti-C3 antibodies [5]. Though the precipitation assay has the least specificity, precipitating also other proteins, the ELISA assays, e.g., the complement-dependent assays, are challenged by the presence of anti-C1q antibodies as well as rheumatoid factor, either blocking or amplifying IC levels. Further, we and others have demonstrated that complement-opsonized ICs are not inflammatory, with C1q signaling through complement receptors such as LAIR-1 to induce silent clearance [2, 10, 26]. Instead, ICs devoid of C1q, that is, signaling through FcγRs, are highly inflammatory, particularly if containing nucleic acid material [2].

Early work in the 1970s investigated IgG binding to neutrophils, capturing both circulating ICs and anti-neutrophil antibodies [6–8]. We here demonstrate that analysis of neutrophil FcγRIIA cell surface expression, and not IgG levels, by flow cytometry accurately captures levels of circulating ICs in patient samples. The advantages with our novel technology are the lack of influence from rheumatoid factor and anti-C1q antibodies and the assessment of “pathogenic” ICs, e.g., ICs signaling through inflammatory FcγR-mediated pathways rather than through complement receptors.

Prior work, pioneered by the group of Lars Ronnblom, clearly demonstrated that nucleic acid-containing ICs, in vitro, promote induction of type I IFNs by plasmacytoid dendritic cells in an FcγRIIA- and TLR-dependent manner [1]. These cytokines are central in SLE pathogenesis, with a majority of the patients having a “type I IFN signature,” in particular, in active disease [27, 28]. To our knowledge, our investigation is the first to demonstrate a direct association between levels of circulating ICs and type I IFN activity in SLE patients. This is important as other inducers of type I IFNs, including cGAMP, a second messenger acting in the cGAS-STING pathway, have recently been implicated in SLE [29]. Further studies are needed to investigate which interferogenic pathways are activated in SLE patients.

Upon IC formation, complement C1q will bind to the Fc region of the IgG antibody and initiate the activation of the classical pathway of the complement system, leading to the consumption of complement components, C3 and C4, and generation of complement split fragments, C3dg, as also demonstrated in our investigation. Complement activation is commonly observed in SLE, with complement activation fragments, in particular, C3a and C5a, acting to recruit inflammatory cells to the area of IC deposition. Upon recruitment, neutrophils will be activated, through FcγRIIA, and release inflammatory and cytotoxic components, including reactive oxygen species (ROS) and proteases, causing tissue damage. Consistent with these mechanisms, levels of ICs, as determined by IC-FLOW, identified patients with complement consumption and, not least, nephritis. These results clearly demonstrate that our novel assay, IC-FLOW, captures events involved in IC-mediated organ damage and inflammation, key processes in lupus pathogenesis. Finally, levels of ICs were associated with disease activity, including the skin, joints, and kidney. Long term, we anticipate IC-FLOW to stratify SLE patients, identifying patients with an IC-driven disease, likely to benefit from B cell-targeted therapy.

## Conclusions

In all, we have developed a novel method, IC-FLOW, to assess levels of circulating ICs in patients with autoimmune diseases, with a focus on SLE. This assay identified SLE patients with an active severe disease, including ongoing nephritis. Of note, the assay performed better than a commercial assay. Future studies aim to determine whether levels of ICs can predict disease progression.

## Data Availability

The data generated or analyzed during this study are available from the corresponding author on reasonable request.

## Abbreviations

IC: Immune complex
IFN: Interferon
PEG: Polyethylene glycol
NETs: Neutrophil extracellular traps
SLE: Systemic lupus erythematosus
TLR: Toll-like receptor
